# *N*,*N*′-Bis­[(*E*)-(6-methyl-2-pyridyl)­methyl­ene]hexane-1,6-diamine

**DOI:** 10.1107/S1600536809016730

**Published:** 2009-05-14

**Authors:** Manuela Ramos Silva, Joana A. Silva, Ana Matos Beja, Abilio J. F.N. Sobral

**Affiliations:** a CEMDRX, Physics Department, University of Coimbra, P-3004-516 Coimbra, Portugal; b Chemistry Department, University of Coimbra, P-3004-516 Coimbra, Portugal

## Abstract

The title compound, C_20_H_26_N_4_, is composed of two (6-methyl-2-pyridyl)methyl­ene units linked by a 1,6-diamine hexane chain. The mol­ecule has *C_i_* symmetry with the inversion center situated at the mid-point of the central C—C bond. The alkyl chain has an all-*trans* conformation, with all the non-H atoms sharing the same plane  [maximum deviation 0.004 (3) Å]. The pyridylmethyl­ene groups are also planar [maximum deviation 0.009 (3) Å], making an angle of 53.78 (19)° with the hexane chain plane. In the crystal, the mol­ecules assemble in layers, stacking along the *a* axis. The stacks are hold together by attractive interactions between π electron systems.

## Related literature

For salen ligands, their structures and possible applications, see: Cozzi (2004 ▶); Li *et al.* (2007 ▶); Renehan *et al.* (2005 ▶); Mohamed *et al.* (2006 ▶). For ruthenium–salen complexes, see: Wu & Gorden (2007 ▶). For the use of salen ligands to form metal-organic frameworks, see: Bu *et al.* (2001 ▶); van den Berga & Arean (2008 ▶).
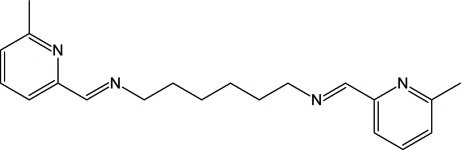


## Experimental

### 

#### Crystal data

C_20_H_26_N_4_
*M* *_r_* = 322.45
Orthorhombic, 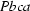

*a* = 7.2713 (10) Å
*b* = 12.6671 (18) Å
*c* = 20.458 (3) Å
*V* = 1884.3 (5) Å^3^
*Z* = 4
Mo *K*α radiation
μ = 0.07 mm^−1^
*T* = 293 K
0.17 × 0.12 × 0.09 mm

#### Data collection

Bruker SMART APEX CCD area-detector diffractometer
Absorption correction: multi-scan (*SADABS*; Sheldrick, 2000 ▶) *T*_min_ = 0.891, *T*_max_ = 0.991
7591 measured reflections
2308 independent reflections
742 reflections with *I* > 2σ(*I*)
*R*_int_ = 0.066

#### Refinement

*R*[*F* ^2^ > 2σ(*F* ^2^)] = 0.056
*wR*(*F* ^2^) = 0.184
*S* = 0.88
2308 reflections
111 parameters
H-atom parameters constrained
Δρ_max_ = 0.12 e Å^−3^
Δρ_min_ = −0.15 e Å^−3^

### 

Data collection: *SMART* (Bruker, 2003 ▶); cell refinement: *SAINT* (Bruker, 2003 ▶); data reduction: *SAINT*; program(s) used to solve structure: *SHELXS97* (Sheldrick, 2008 ▶); program(s) used to refine structure: *SHELXL97* (Sheldrick, 2008 ▶); molecular graphics: *ORTEPII* (Johnson, 1976 ▶); software used to prepare material for publication: *SHELXL97*.

## Supplementary Material

Crystal structure: contains datablocks global, I. DOI: 10.1107/S1600536809016730/su2109sup1.cif

Structure factors: contains datablocks I. DOI: 10.1107/S1600536809016730/su2109Isup2.hkl

Additional supplementary materials:  crystallographic information; 3D view; checkCIF report

## Figures and Tables

**Fig. 1.**
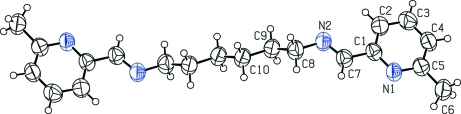
ORTEPII (Johnson, 1976) plot of the title compound. Displacement ellipsoids are drawn at the 50% probability level.

**Fig. 2.**
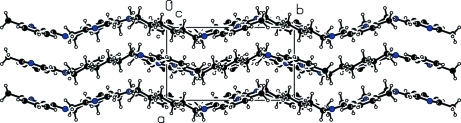
Crystal packing of the title compound viewed along the c axis.

## supplementary crystallographic information

### Comment

Schiff bases and their complexes (salen ligands) continue to raise interest, even after a hundred years of research, due to their novel structures, their application in reversible binding of oxygen, their catalytic activity in hydrogenation of olefins, intermolecular transfer of amino groups, and their complexing ability towards some toxic metals (Cozzi, 2004; Li *et al.*, 2007; Renehan *et al.*; 2005, Mohamed *et al.*, 2006). Two important examples are, copper(I)-salen complexes investigated as antitumor agents, and ruthenium-salen complexes studied as protein kinase inhibitors by mimicking the structure of organic indolocarbazoles (Wu & Gorden, 2007). Salen complexes have also been used to form metal-organic frameworks (MOFs), which are intensively sought for the storage of hydrogen and carbon dioxide (Berga & Arean, 2008).

The title compound was synthesized to be used as a ligand/spacer in the construction of MOFs. For such purposes long-chain bidentate ligands may be useful to alter the cavity size, as reported by Bu *et al.*, who showed that in some Cu(II) coordination compounds, the cavity size depends on the chain length of bis-sulfinyl ligands used.

The title compound is illustrated in Fig. 1, and the geometrical parameters are available in the archived CIF. It crystallizes with half a molecule in the asymmetric unit. The center of inversion is located at the middle point of the alkyl chain (C10-C10a). The hexane chain adopts an all-*trans* conformation. The mean plane of the pyridylmethylene group makes an angle of 53.78 (19)° with the central chain plane. The short C7–N2 bond length of 1.257 (3) Å, shows the double bond character of this bond.

In the crystal structure the molecules assemble in layers stacked along the *a* axis, as shown in Fig. 2.

### Experimental

5.5 mmol of 1,6-diamine was added to 11 mmol of 6-methyl-pyridil-2-aldehyde in toluene (50 ml). The mixture was stirred at 160°C with reflux in a Dean-Stark system until all the water was removed (~2 h). The solution was washed with diluted HCl (30 ml) and NaHCO_3_ (15 ml) and dried with NaSO_4_ anhydrous (5 g). Solvent was evaporated in a stirring water bath at 40°C under nitrogen. The product was recrystallized from CH_2_Cl_2_ to give the title compound in 40% yield.

### Refinement

The crystals of the title compound diffracted very poorly, displaying broad weak reflections, hence the ratio of observed/unique reflections is only 32%. H-atoms were positioned geometrically [C-H = 0.93 - 0.97 Å] and refined using a riding model [U_iso_(H) = 1.2 or 1.5U_eq_(parent C-atom)].

### Crystal data

**Table:**

C_20_H_26_N_4_	*F*(000) = 696
*M**_r_* = 322.45	*D*_x_ = 1.137 Mg m^−^^3^
Orthorhombic, *P**b**c**a*	Mo *K*α radiation, λ = 0.71073 Å
Hall symbol: -P 2ac 2ab	Cell parameters from 546 reflections
*a* = 7.2713 (10) Å	θ = 3.2–20.3°
*b* = 12.6671 (18) Å	µ = 0.07 mm^−^^1^
*c* = 20.458 (3) Å	*T* = 293 K
*V* = 1884.3 (5) Å^3^	Prism, yellow
*Z* = 4	0.17 × 0.12 × 0.09 mm

### Data collection

**Table:**

Bruker APEX CCD area-detector diffractometer	2308 independent reflections
Radiation source: fine-focus sealed tube	742 reflections with *I* > 2σ(*I*)
graphite	*R*_int_ = 0.066
φ and ω scans	θ_max_ = 28.3°, θ_min_ = 2.0°
Absorption correction: multi-scan (*SADABS*; Sheldrick, 2000)	*h* = −9→8
*T*_min_ = 0.891, *T*_max_ = 0.991	*k* = −16→12
7591 measured reflections	*l* = −22→25

### Refinement

**Table:**

Refinement on *F*^2^	Primary atom site location: structure-invariant direct methods
Least-squares matrix: full	Secondary atom site location: difference Fourier map
*R*[*F*^2^ > 2σ(*F*^2^)] = 0.056	Hydrogen site location: inferred from neighbouring sites
*wR*(*F*^2^) = 0.184	H-atom parameters constrained
*S* = 0.88	*w* = 1/[σ^2^(*F*_o_^2^) + (0.0774*P*)^2^] where *P* = (*F*_o_^2^ + 2*F*_c_^2^)/3
2308 reflections	(Δ/σ)_max_ < 0.001
111 parameters	Δρ_max_ = 0.12 e Å^−^^3^
0 restraints	Δρ_min_ = −0.14 e Å^−^^3^

### Special details

**Table:**

Geometry. All e.s.d.'s (except the e.s.d. in the dihedral angle between two l.s. planes) are estimated using the full covariance matrix. The cell e.s.d.'s are taken into account individually in the estimation of e.s.d.'s in distances, angles and torsion angles; correlations between e.s.d.'s in cell parameters are only used when they are defined by crystal symmetry. An approximate (isotropic) treatment of cell e.s.d.'s is used for estimating e.s.d.'s involving l.s. planes.
Refinement. Refinement of *F*^2^ against ALL reflections. The weighted *R*-factor *wR* and goodness of fit *S* are based on *F*^2^, conventional *R*-factors *R* are based on *F*, with *F* set to zero for negative *F*^2^. The threshold expression of *F*^2^ > σ(*F*^2^) is used only for calculating *R*-factors(gt) *etc*. and is not relevant to the choice of reflections for refinement. *R*-factors based on *F*^2^ are statistically about twice as large as those based on *F*, and *R*- factors based on ALL data will be even larger.

### Fractional atomic coordinates and isotropic or equivalent isotropic displacement parameters (Å^2^)

**Table:**

	*x*	*y*	*z*	*U*_iso_*/*U*_eq_	
N2	0.1193 (3)	0.29540 (19)	0.10261 (10)	0.0731 (7)	
C1	0.0485 (4)	0.4665 (2)	0.14588 (13)	0.0633 (8)	
N1	−0.0101 (3)	0.56245 (18)	0.12827 (10)	0.0667 (7)	
C5	−0.0300 (4)	0.6357 (2)	0.17502 (13)	0.0683 (8)	
C10	0.0506 (4)	0.05151 (18)	−0.00364 (12)	0.0711 (8)	
H10A	0.0034	0.0880	−0.0418	0.085*	
H10B	0.1796	0.0366	−0.0115	0.085*	
C9	0.0355 (4)	0.1241 (2)	0.05473 (12)	0.0719 (8)	
H9A	−0.0932	0.1400	0.0624	0.086*	
H9B	0.0820	0.0877	0.0930	0.086*	
C2	0.0901 (4)	0.4398 (2)	0.20945 (14)	0.0792 (9)	
H2	0.1312	0.3724	0.2199	0.095*	
C7	0.0688 (4)	0.3890 (2)	0.09307 (13)	0.0674 (8)	
H7	0.0430	0.4102	0.0505	0.081*	
C8	0.1391 (4)	0.2259 (2)	0.04644 (12)	0.0760 (9)	
H8A	0.2684	0.2104	0.0399	0.091*	
H8B	0.0945	0.2619	0.0077	0.091*	
C6	−0.0943 (5)	0.7425 (2)	0.15397 (13)	0.0892 (10)	
H6A	0.0012	0.7933	0.1616	0.134*	
H6B	−0.2018	0.7617	0.1785	0.134*	
H6C	−0.1238	0.7411	0.1082	0.134*	
C4	0.0072 (4)	0.6140 (2)	0.23978 (14)	0.0762 (9)	
H4	−0.0095	0.6658	0.2715	0.091*	
C3	0.0690 (4)	0.5156 (3)	0.25715 (14)	0.0852 (10)	
H3	0.0963	0.5002	0.3005	0.102*	

### Atomic displacement parameters (Å^2^)

**Table:**

	*U*^11^	*U*^22^	*U*^33^	*U*^12^	*U*^13^	*U*^23^
N2	0.0935 (18)	0.0568 (16)	0.0689 (14)	−0.0005 (13)	−0.0027 (13)	−0.0059 (12)
C1	0.071 (2)	0.061 (2)	0.0577 (16)	−0.0117 (15)	0.0005 (15)	−0.0023 (14)
N1	0.0852 (17)	0.0543 (15)	0.0605 (13)	−0.0067 (13)	0.0013 (12)	−0.0032 (12)
C5	0.080 (2)	0.064 (2)	0.0614 (17)	−0.0082 (16)	−0.0006 (16)	−0.0020 (15)
C10	0.091 (2)	0.0550 (18)	0.0675 (16)	0.0042 (15)	0.0070 (17)	0.0000 (14)
C9	0.091 (2)	0.0575 (18)	0.0677 (17)	0.0042 (17)	0.0051 (16)	−0.0029 (14)
C2	0.101 (3)	0.067 (2)	0.0701 (19)	−0.0010 (18)	−0.0052 (18)	−0.0026 (17)
C7	0.081 (2)	0.061 (2)	0.0605 (16)	−0.0091 (16)	−0.0001 (15)	−0.0005 (15)
C8	0.097 (2)	0.065 (2)	0.0654 (17)	0.0011 (17)	0.0064 (16)	−0.0070 (15)
C6	0.130 (3)	0.061 (2)	0.0771 (18)	0.0014 (19)	0.000 (2)	−0.0048 (16)
C4	0.095 (2)	0.068 (2)	0.0651 (19)	−0.0068 (19)	−0.0056 (16)	−0.0116 (15)
C3	0.114 (3)	0.081 (2)	0.0602 (17)	−0.004 (2)	−0.0119 (18)	0.0020 (18)

### Geometric parameters (Å, °)

**Table:**

N2—C7	1.257 (3)	C9—H9A	0.9700
N2—C8	1.455 (3)	C9—H9B	0.9700
C1—N1	1.338 (3)	C2—C3	1.377 (4)
C1—C2	1.377 (4)	C2—H2	0.9300
C1—C7	1.467 (4)	C7—H7	0.9300
N1—C5	1.340 (3)	C8—H8A	0.9700
C5—C4	1.380 (4)	C8—H8B	0.9700
C5—C6	1.495 (4)	C6—H6A	0.9600
C10—C10^i^	1.506 (5)	C6—H6B	0.9600
C10—C9	1.511 (3)	C6—H6C	0.9600
C10—H10A	0.9700	C4—C3	1.373 (4)
C10—H10B	0.9700	C4—H4	0.9300
C9—C8	1.503 (3)	C3—H3	0.9300
			
C7—N2—C8	118.5 (2)	C1—C2—H2	120.9
N1—C1—C2	123.2 (3)	N2—C7—C1	123.1 (3)
N1—C1—C7	116.2 (2)	N2—C7—H7	118.5
C2—C1—C7	120.6 (3)	C1—C7—H7	118.5
C1—N1—C5	118.1 (2)	N2—C8—C9	112.4 (2)
N1—C5—C4	121.7 (3)	N2—C8—H8A	109.1
N1—C5—C6	117.1 (2)	C9—C8—H8A	109.1
C4—C5—C6	121.2 (3)	N2—C8—H8B	109.1
C10^i^—C10—C9	114.4 (3)	C9—C8—H8B	109.1
C10^i^—C10—H10A	108.7	H8A—C8—H8B	107.9
C9—C10—H10A	108.7	C5—C6—H6A	109.5
C10^i^—C10—H10B	108.7	C5—C6—H6B	109.5
C9—C10—H10B	108.7	H6A—C6—H6B	109.5
H10A—C10—H10B	107.6	C5—C6—H6C	109.5
C8—C9—C10	113.3 (2)	H6A—C6—H6C	109.5
C8—C9—H9A	108.9	H6B—C6—H6C	109.5
C10—C9—H9A	108.9	C3—C4—C5	119.6 (3)
C8—C9—H9B	108.9	C3—C4—H4	120.2
C10—C9—H9B	108.9	C5—C4—H4	120.2
H9A—C9—H9B	107.7	C4—C3—C2	119.1 (3)
C3—C2—C1	118.3 (3)	C4—C3—H3	120.5
C3—C2—H2	120.9	C2—C3—H3	120.5
			
C2—C1—N1—C5	−0.4 (4)	N1—C1—C7—N2	−178.7 (3)
C7—C1—N1—C5	−179.8 (2)	C2—C1—C7—N2	1.9 (4)
C1—N1—C5—C4	−0.3 (4)	C7—N2—C8—C9	−127.9 (3)
C1—N1—C5—C6	179.6 (3)	C10—C9—C8—N2	178.8 (2)
C10^i^—C10—C9—C8	179.5 (3)	N1—C5—C4—C3	1.0 (4)
N1—C1—C2—C3	0.4 (5)	C6—C5—C4—C3	−178.9 (3)
C7—C1—C2—C3	179.9 (3)	C5—C4—C3—C2	−0.9 (5)
C8—N2—C7—C1	−178.5 (2)	C1—C2—C3—C4	0.3 (5)

Symmetry codes: (i) −*x*, −*y*, −*z*.

## References

[bb1] Berga, A. W. C. van den & Arean, C. O. (2008). *Chem. Commun.* pp. 668–681. 10.1039/b712576n 18478688

[bb2] Bruker (2003). *SMART* and *SAINT* Bruker AXS Inc., Madison, Wisconsin, USA.

[bb3] Bu, X.-H., Chen, W., Lu, S.-L., Zhang, R.-H., Liao, D.-Z., Bu, W.-M., Shionoya, M., Brisse, F. & Ribas, J. (2001). *Angew. Chem. Int. Ed* **40**, 3201–3203. 10.1002/1521-3773(20010903)40:17<3201::AID-ANIE3201>3.0.CO;2-Z 29712046

[bb4] Cozzi, P. G. (2004). *Chem. Soc. Rev.* **33**, 410–421.

[bb5] Johnson, C. K. (1976). *ORTEPII* Report ORNL-5138. Oak Ridge National Laboratory, Tennessee, USA.

[bb6] Li, Y.-G., Shi, D.-H., Zhu, H. & Ng, S. W. (2007). *Inorg. Chim. Acta*, **360**, 2881–2889.

[bb7] Mohamed, G. G., Omar, M. M. & Hindy, A. M. (2006). *Turk J. Chem.* **30**, 361–382.

[bb8] Renehan, M. F., Schanz, H.-J., McGarridge, E. M., Dalton, C. T., Daly, A. M. & Gilhuany, D. G. (2005). *J. Mol. Catal. A*, **231**, 205–220.

[bb9] Sheldrick, G. M. (2000). *SADABS* University of Göttingen, Germany.

[bb10] Sheldrick, G. M. (2008). *Acta Cryst.* A**64**, 112–122. 10.1107/S0108767307043930 18156677

[bb11] Wu, X. & Gorden, A. E. V. (2007). *J. Comb. Chem.* **9**, 601–608. 10.1021/cc070021q 17497932

